# Supplementary figures and images for: The Effect of Sera from Children with Obstructive Sleep Apnea Syndrome (OSAS) on Human Cardiomyocytes Differentiated from Human Embryonic Stem Cells
Source: Int J Mol Sci. 2021 Oct 22;22(21):11418. doi: 10.3390/ijms222111418 (PMC8584070; doi:10.3390/ijms222111418)

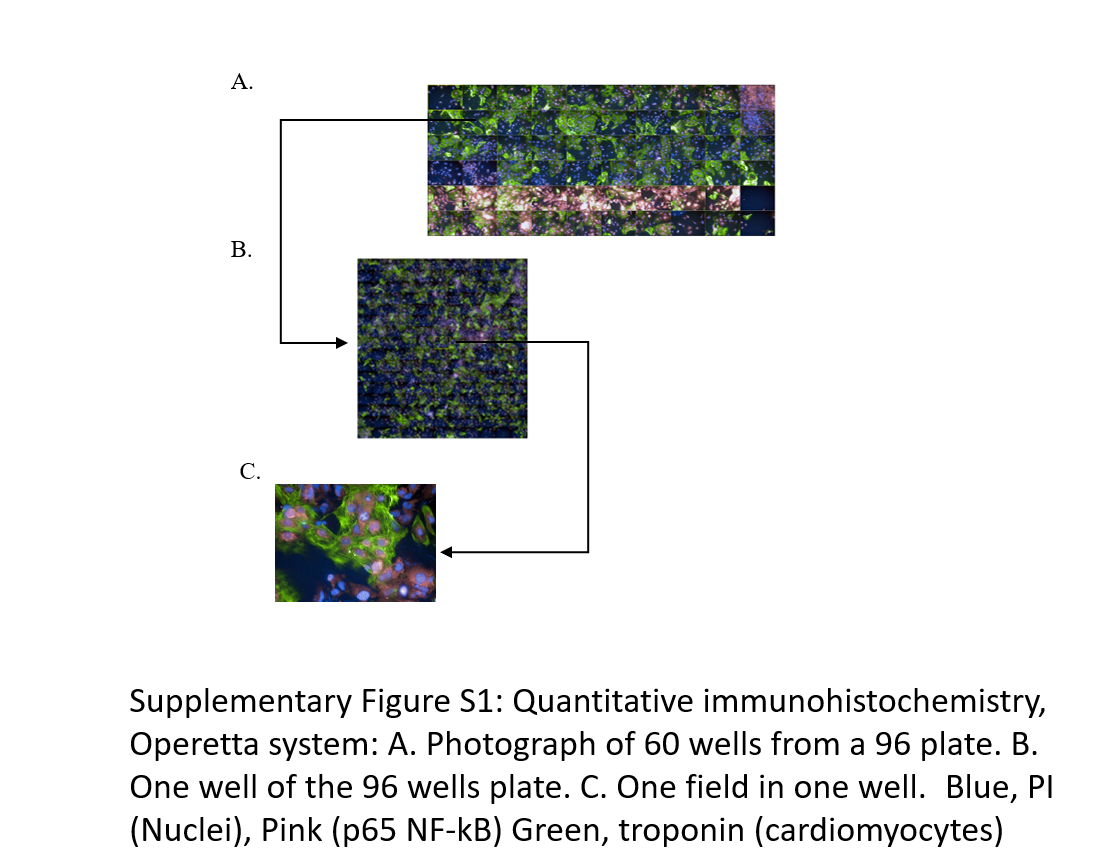

Supplement: Supplementary file 1 [file ijms-22-11418-s001.zip › ijms-1370447-supplementary.png]
